# Effects of Age, Phase Variation and Pheromones on Male Sperm Storage in the Desert Locust, *Schistocerca gregaria*

**DOI:** 10.3390/insects12070642

**Published:** 2021-07-14

**Authors:** Satoshi Hiroyoshi, Takayuki Mitsunaga, Tomoko Ganaha-Kikumura, Gadi V. P. Reddy

**Affiliations:** 1 Department of Chemical Ecology, International Centre of Insect Physiology and Ecology (ICIPE), Nairobi P.O. Box 30772-00100, Kenya; 2 Independent Researcher, Kawagoe 350-1115, Saitama, Japan; 3 Institute of Plant Protection National Agriculture and Food Research Organization, 2-1-18 Kannondai, Tsukuba 305-8602, Ibaraki, Japan; aeiou@affrc.go.jp; 4 Okinawa Prefectural Central Wholesale Market, Okinawa 901-2128, Okinawa, Japan; kikumurt@pref.okinawa.lg.jp; 5 USDA-ARS-Southern Insect Management Research Unit, 141 Experiment Station Road, P.O. Box 346, Stoneville, MS 38776, USA; gadi.reddy@usda.gov

**Keywords:** reproduction, seminal vesicle, sperm, testis, vas deferens

## Abstract

**Simple Summary:**

We investigated male sperm storage in the desert locust *Schistocerca gregaria*. Phase (solitary or gregarious) did not affect sperm distribution in the vas deferens and seminal vesicle, whereas sperm accumulation of the seminal vesicle in gregarious locusts was promoted more than in solitary ones. Pheromones received from neither mature adults nor nymphs affected sperm distribution in the vas deferens and seminal vesicle. However, sperm accumulation in the seminal vesicle was more promoted in the gregarious locusts which received pheromones from mature adults than those obtained from nymphs at early adult stage, especially seven days after adult emergence.

**Abstract:**

In general, sperm produced in the testis are moved into the seminal vesicle via the vas deferens in insects, where they are stored. How this sperm movement is controlled is less well understood in locusts or grasshoppers. In this study, the effects of age, phase variation and pheromones on male sperm storage were investigated in the desert locust, *Schistocerca gregaria* (Forskål). In this locust, a pair of ducts, the vasa deferentia, connect the testes to a pair of the long, slender seminal vesicles that are folded approximately thirty times, and where the sperm are stored. We found that phase variation affected the level of sperm storage in the seminal vesicle. Moreover, adult males that detected pheromones emitted by mature adult males showed enhanced sperm storage compared with males that received the pheromones emitted from nymphs: The former, adult male pheromones are known to promote sexual maturation of immature adults of both sexes, whereas the latter, nymphal pheromones delay sexual maturation. Most mature adult males had much sperm in the vasa deferentia at all times examined, suggesting daily sperm movement from the testes to the seminal vesicles via the vasa deferentia. As adult males aged, sperm were accumulated from the proximal part to the distal end of the seminal vesicle. Many sperm remained in the seminal vesicle after mating. These results suggest that young or new sperm located near the proximal part of the seminal vesicle could be used for mating, whereas old sperm not used for mating are stored in the distal part of the seminal vesicle.

## 1. Introduction

The desert locust, *Schistocerca gregaria* (Forskål) (Orthoptera: Acrididae), distributed from Africa to parts of western Asia, is a notorious pest, associated with human famines. This locust has phase variation associated with group behavior changes and long-distance migration [1,2,3,4,5,6,7]. Previous studies of this locust have examined male reproduction, i.e., mating and sexual behavior [8,9,10,11], accessory glands [12,13,14,15,16], sperm transfer [17,18,19], testes development [20,21], and spermatogenesis [22,23,24,25,26]. The development of this locust is regulated by several abiotic factors (temperature, relative humidity, and diet), and biotic factors such as locust density, paternal and maternal effects, age, pheromones, tactile or visual stimulus, hormones, and phase variation [27,28,29,30,31,32,33,34,35]. For example, pheromones emitted from mature adult males promote sexual maturation of immature adults of both sexes, and earlier copulative behavior and integumental yellowing of males, compared to controls [36,37,38,39,40,41,42]. In contrast, nymphal pheromones delay the reproductive development of immature adults of both sexes, with adult males requiring almost one month of sexual development before mating [27,37,38,43]. These two pheromones are called maturation accelerating and maturation retarding pheromones, respectively [6]. These pheromones are useful to synchronize the development of population. Moreover, the desert locust undergoes phase variation, with solitary and gregarious phases. These phase morphs show different body coloration, behavior, development, reproduction, and morphology [6,44,45,46]. Pheromone production and sexual maturation in the solitary phase are delayed in comparison to gregarious phase locusts [6]. However, the sperm supply system, which consists of spermatogenesis, sperm storage, and ejaculation, is modulated in desert locusts, and how male sperm storage changes due to pheromones and phase variation are unclear.

The male desert locust has internal reproductive organs whose structure is similar to that in other locusts or grasshoppers. The locusts have large testes, a couple of vasa deferentia, 15 paired accessory glands, a pair of seminal vesicles, and an ejaculatory duct [47,48,49]. The seminal vesicle of the desert locust is long and slender, and is folded approximately thirty times. It opens into the ejaculatory duct and the vasa deferentia. The sperm are supplied by the testis via the vasa deferentia, and must exit through the seminal vesicles during mating. How the sperm are accumulated in the seminal vesicle is unclear in the desert locust and other Orthoptera. In the present study, we examined the pattern of male sperm storage in the vas deferens and seminal vesicle with aging. We also assessed the effects of phase variation and pheromones on male sperm storage in the seminal vesicle.

## 2. Materials and Methods

### 2.1. Insects

*Schistocerca gregaria* used in the experiments were reared in an insectary at the International Centre of Insect Physiology and Ecology (ICIPE) in Nairobi, Kenya, under gregarious conditions, except for locusts in the solitary phase, using procedures described by Rai et al. [50], except for rearing temperatures. In brief, locusts (300–400) of both sexes were bred in aluminum cages (50 × 50 × 50 cm). After ecdysis to the adult stage, locusts were collected from the stock colonies and were transferred to other rooms maintained at 30 ± 2 °C (phase variation research) or 32 ± 2 °C (pheromonal research) and a photoperiod of L12: D12. Adults treated with pheromones were reared separately in a different room from other locusts to avoid contamination with pheromones. Adult males were kept in aluminum cages (15.5 × 15.5 × 31 cm height) and fed daily on a diet of wheat bran and wheat seedlings. Solitary locusts from an isolated-reared-line were reared individually in aluminum cages (10 × 12 × 14 cm height) throughout their life except for the egg stage, well separated from the rearing room with gregarious phase locusts.

### 2.2. Sperm Storage

**Figure 1:**
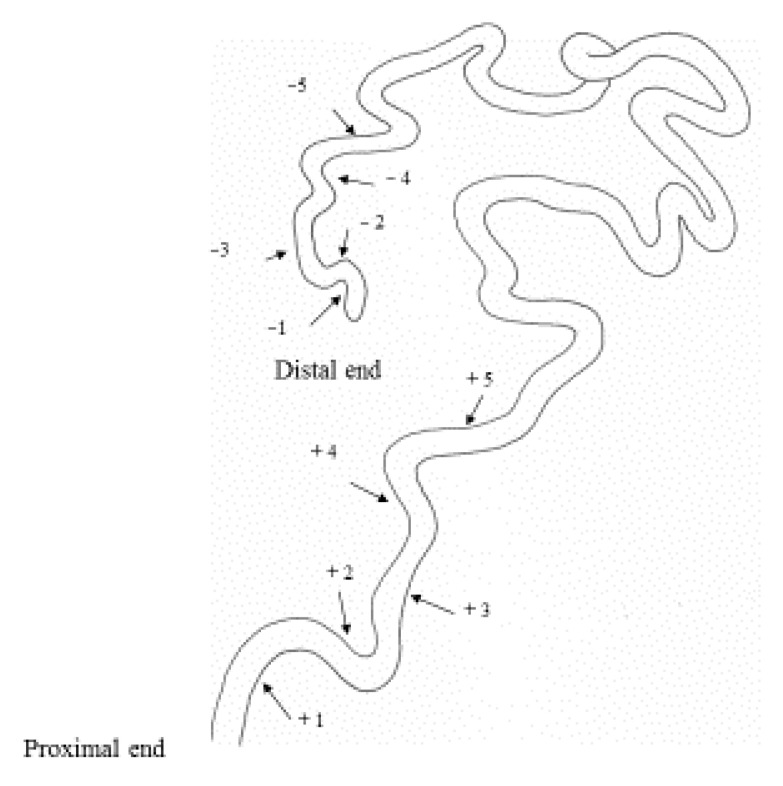
Structure of the seminal vesicle in the desert locust, *Shistocerca gregaria*. Numbering was made from the proximal end (+1, +2, +3...) and the distal end (−1, −2, −3…) of the seminal vesicle.

To examine the effects of age, phase variation, or pheromone emission on sperm storage, male locusts were dissected in 0.86% NaCl solution at the desired age, and the vas deferens and seminal vesicle were removed from other reproductive organs using a pair of forceps. As the number of intricately intertwined sperm could not be counted accurately, the quantity of sperm was estimated by the distribution of sperm in the seminal vesicle. Most sperm in the seminal vesicle occurred as bundles (spermatodesms) [51]. The number of sperm was classified into four categories by observing each fold region of the seminal vesicle under a microscope as follows; 0 = empty, 1 = several free sperm and/or <10 sperm bundles are seen in the fold (a few), 2 = ≥10 dispersed sperm bundles and/or intermittent sperm bundles mass (common), and 3 = many sperm bundles are packed tightly in the fold (many). In preliminary studies, we found that the total number of fold regions in the seminal vesicle varied among individuals. The first 10 folds of the vesicle, at both the vesicle’s beginning and end, were marked for examination, with those at the proximal end being 1 to 10 and those at the distal end being −1 to −10 (see Figure 1). Using these numbered regions as reference points, the presence or absence of sperm in the vasa deferentia and seminal vesicles was observed in adults of various ages. 

### 2.3. Phase Variation

To compare sperm storage between locusts in the solitary and gregarious phases, solitary virgin male locusts were individually reared as mentioned above. In contrast, the gregarious virgin locusts were collectively reared in aluminum cages (15.5 × 15.5 × 31 cm height) in groups of approximately five male individuals at 30 ± 2 °C from adult emergence. Locusts were dissected at 0, 7, 14, or 28 days after adult emergence.

In this experiment, three sets of observations were made. First, the presence or absence of sperm in the vasa deferentia and seminal vesicles was determined in solitary versus gregarious male locusts. Second, the folds in right side of the seminal vesicle were counted in locusts of each phase. Third, the sperm distribution among folds of right side of the seminal vesicle was also examined. For the first two experiments, the sample size differed among each point. For the last observation, the sample size was ten locusts for each point. Although the sample size was different, each of the three data were obtained from the same individual.

### 2.4. Effect of Pheromone

To investigate the effect of pheromones on sperm storage, five newly molted immature gregarious virgin adult males (referred to as recipients) were exposed to five mature (yellow) gregarious virgin adult males or five gregarious nymphal (4th or 5th) males (referred to as donors) in aluminum cages (15.5 × 15.5 × 31 cm height) at 32 ± 2 °C, which allowed the insects to receive only olfactory signals from the donors. The donors were kept in the top part of the cage, whereas the recipients were maintained in the bottom part of the cage. There was an aluminum barrier with small holes between the top and bottom parts in cages, in which only olfactory signals could affect their physiology. When a donor insect died during the exposure period, it was replaced by another donor to keep their density constant. When the donor insects (nymphs) that were the source of the nymphal pheromone molted to the adult stage, they were replaced by a new nymph. Three sets of observations were made; first, the presence or absence of sperm in both sides of the vasa deferentia and seminal vesicles was examined in these two types of male locusts. Second, the number of folds in right side of the seminal vesicle were counted for each type of locust. Third, the sperm distribution in each numbered region (specific fold, as described above), on right side of the seminal vesicle was examined in 10 male locusts at each age and treatment. The recipient locusts were dissected 1, 2, or 4 weeks after exposure to pheromone treatment. Sperm distribution in the vas deferens and seminal vesicle was also observed for 3-day-old locusts.

### 2.5. Statistics

Ordinary logistic regression was used to compare the indexes of levels of stored sperm due to the main effect, days after treatment (DAT), or the differences from the root or the edge from the proximal and distal ends of the seminal vesicle at the marked folds and their interactions.

We used Mann–Whitney’s *U*-tests to compare the length of the seminal vesicle between immature and mature adult males and the number of folds in the seminal vesicle between solitary and gregarious locusts.

## 3. Results

### 3.1. Change Over Time in Sperm Storage

Sperm were found in the vasa deferentia or the seminal vesicles of gregarious male locusts five days (at 30 °C) or three days (at 32 °C) after adult emergence; however, in some individuals kept at 30 °C, there were no sperm in either the vasa deferentia nor the seminal vesicles seven days after adult emergence. These findings indicate that the start of sperm movement differed among individuals and temperatures.

In general, the amount of sperm in the vas deferens seemed to increase with sexual maturation, but the accumulation rate was not quantified in this study. In immature adults, there were usually limited sperm in the vas deferens; excluding adults <10 days old, most solitary (88.9%) and gregarious (94.1%) adults had sperm in their vas deferens (Table 1). Phase variation did not affect the start of sperm migration, nor its storage in the vasa deferentia and seminal vesicles (Table 2).

The length of the seminal vesicle increased with age, and the length in gregarious adults <7 days of age (22.2 ± 2.86 mm SD, n = 8) was significantly shorter than that in the mature gregarious adults (28 days old) (27.6 ± 2.42 mm, n = 6) (*U1*-Value = 3.0, *U2*-Value = 45.0, *W*-value = 66.0, *p* = 0.0047).

### 3.2. Effects of Phase Variation on Sperm Storage

First, in solitary locusts, the number of folds in the seminal vesicle was significantly higher than in the gregarious locusts at each of observed ages (Day 0: *U1*-Value = 53.0, *U2*-Value = 7.0, *W*-Value = 28.0, *p* = 0.0110; Day 7: *U1*-Value = 87.5, *U2*-Value = 22.5, *W*-Value = 77.5, *p* = 0.0217; and Day 14: *U1*-Value = 62.0, *U2*-Value = 8.0, *W*-Value = 36.0, *p* = 0.0068) (Table 3). At Day 28, the differences were marginally not significant (*U1*-Value = 69.5, *U2*-Value = 20.5, *W*-Value = 41.5, *p* = 0.0548).

**Figure 2:**
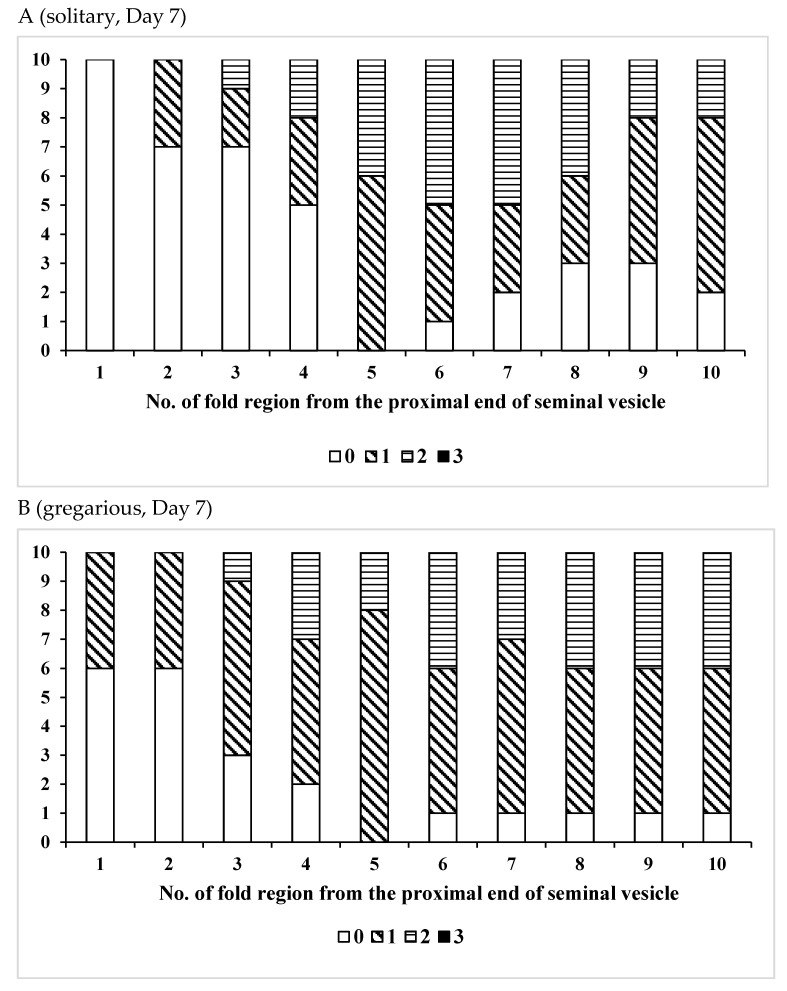

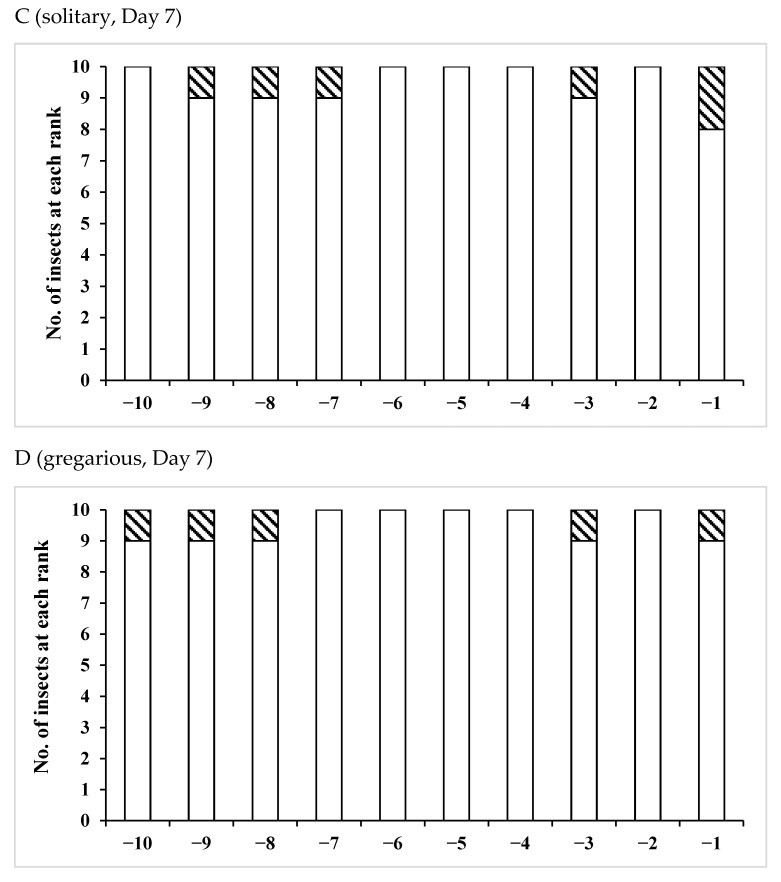

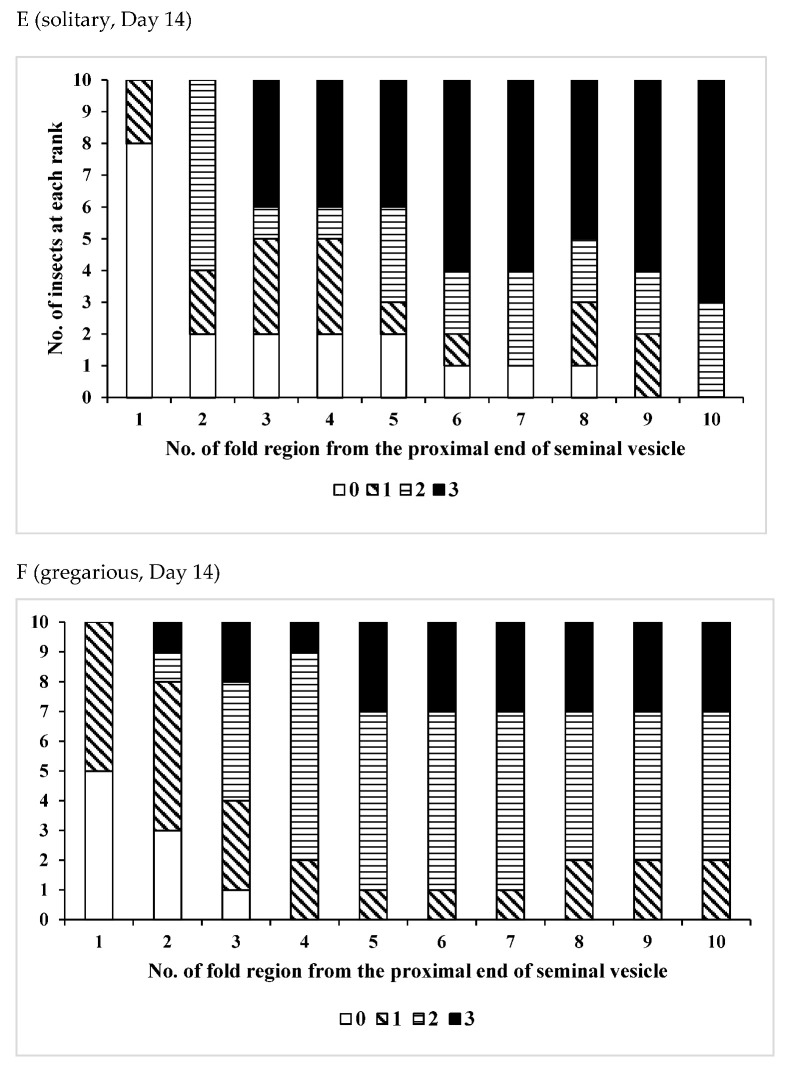

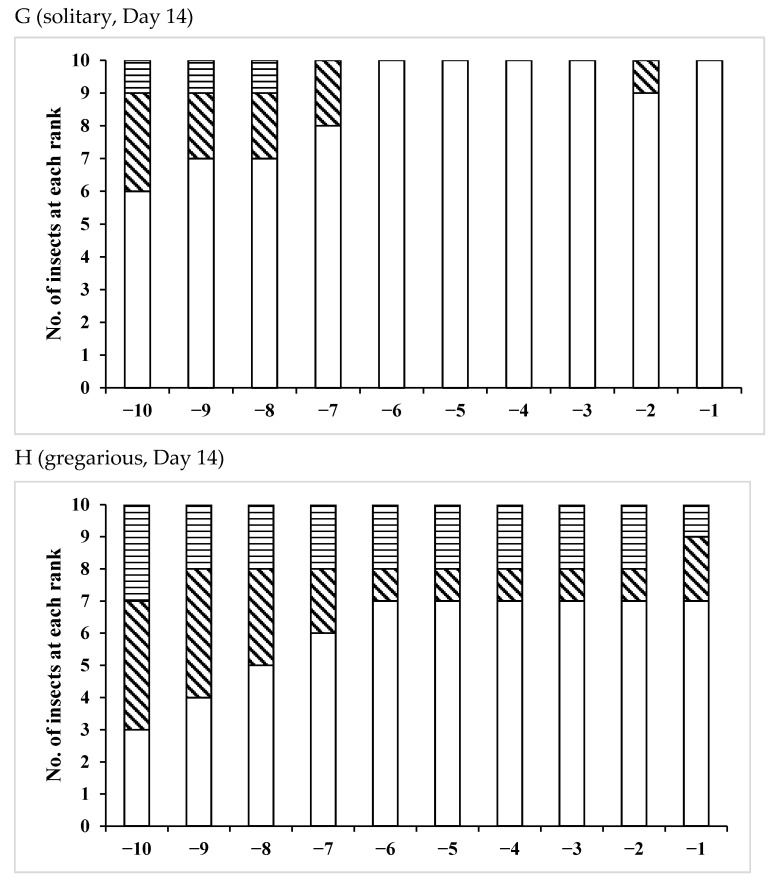

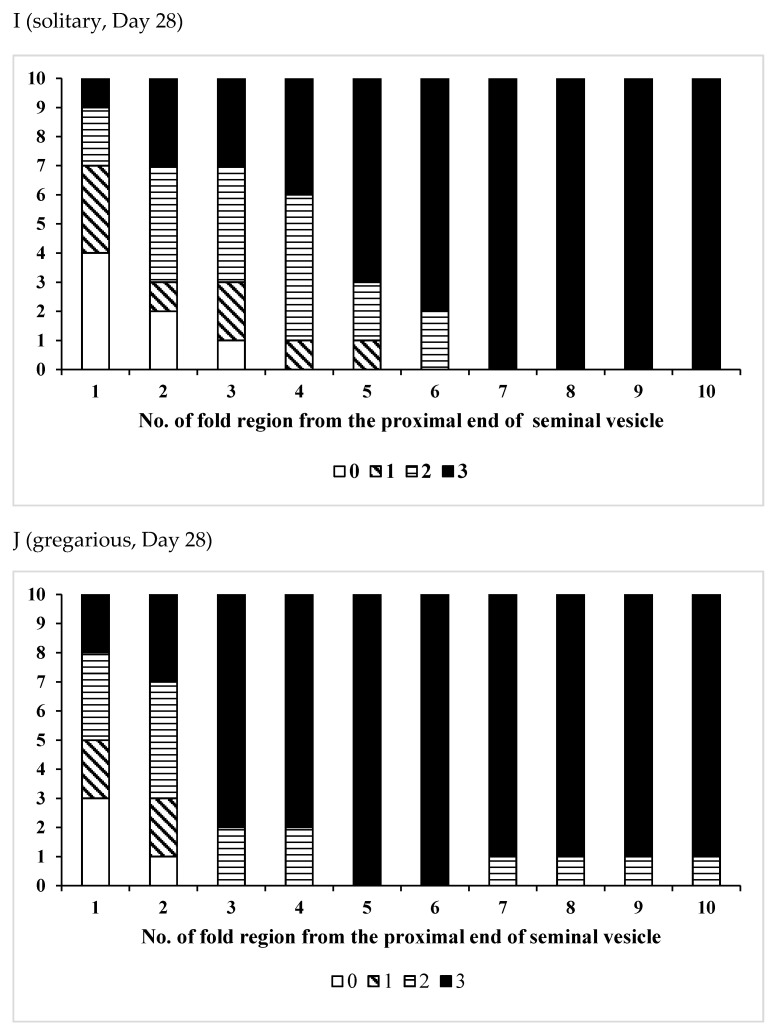

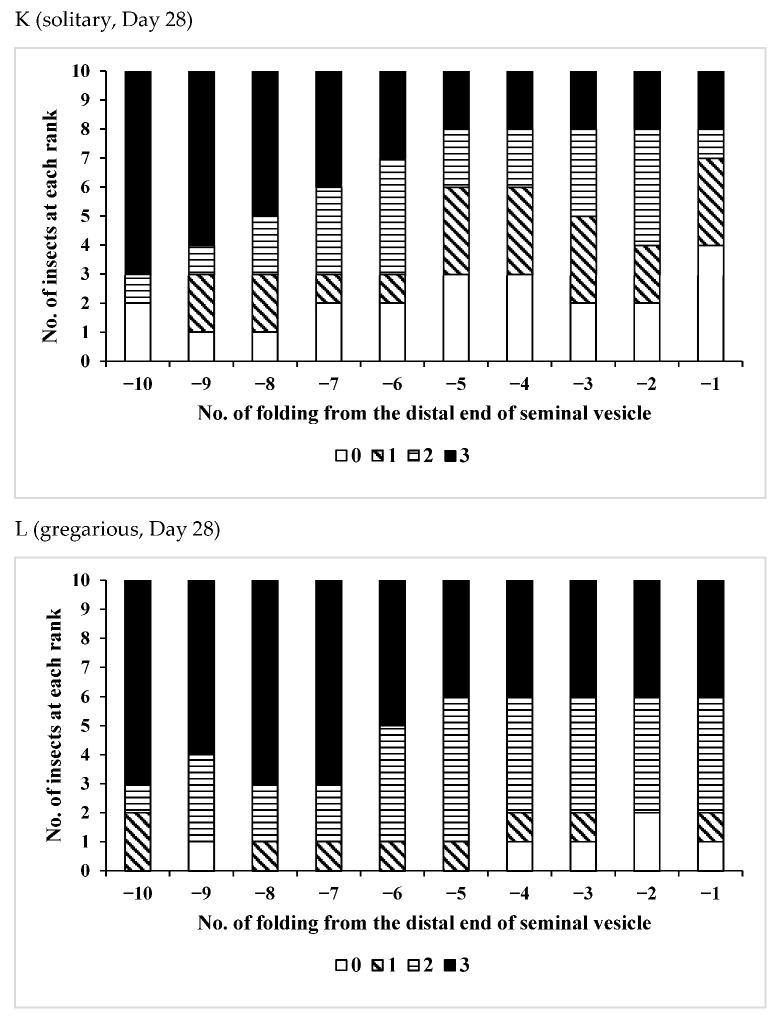
Distribution of sperm in the proximal part (**A**,**E**,**I**) or the distal part (**C**,**G**,**K**) of the seminal vesicle in the solitary form, and that in the proximal part (**B**,**F**,**J**) or the distal part (**D**,**H**,**L**) of the seminal vesicle in the gregarious form of the desert locust, *Shistocerca gregaria*. (**A**–**D**), (**E**–**H**), and (**I**–**L**) indicate Day 7, 14, and 28 of adult life, respectively. The number of sperm was classified into four categories by observing each fold region of the seminal vesicle under a microscope as follows; 0 = empty, 1 = several free sperm and/or <10 sperm bundles are seen in the fold (a few), 2 = ≥10 dispersed sperm bundles and/or intermittent sperm bundles mass (common), and 3 = many sperm bundles are packed tightly in the fold (many).

Second, we investigated spatial storage of sperm in the seminal vesicle in the solitary locusts, and we just found a few sperm in the proximal end (fold regions +1 to +3) of the seminal vesicle, at age 1 week after adult emergence (Figure 2A). Additionally, in this period, there were almost no sperm in the distal part of the seminal vesicle in most individuals (Figure 2C). At 2 weeks after adult emergence, many sperm masses (Categories 2 and 3) were found in the +6 to +10th fold regions in the proximal part of the seminal vesicle for 60 to 70% of all individuals (Figure 2E). At this same time point, there were few sperm in the distal region (−7 to −1st fold regions) of the seminal vesicle (Figure 2G). Four weeks after adult emergence, many sperm masses had accumulated in the proximal part (+5 to +10th fold regions) of the seminal vesicle in 70 to 100% individuals (Figure 2I). At 4 weeks after adult emergence, 10–40% of individuals had no sperm in the distal part of the seminal vesicle (Figure 2K).

Third, we assessed sperm storage in the gregarious locusts, and we found few or no sperm in the proximal part (+1 to +7th fold regions) of the seminal vesicle for 60–100% of males one week after adult emergence (Figure 2B). Additionally, at 1 week, there were no sperm, or almost none, in the seminal vesicle’s distal part (Figure 2D). At 2 weeks after adult emergence, there were many sperm or sperm bundles in the proximal part of the seminal vesicle (except for the first two fold regions) in 20 to 90% of males (Figure 2F), but at 2 weeks, most males (70%) had no sperm in the distal part (−6 to −1st fold regions) of the seminal vesicle (Figure 2H). At 4 weeks after adult emergence, there were many sperm bundles in the proximal part (+3 to +10th fold regions) of the seminal vesicle (Figure 2J), while in the distal portion of the vesicle, most (80 to 90%) males had many sperm or sperm bundles (Figure 2L).

Comparing solitary and gregarious males, we found that as males of both phases aged, sperm in the seminal vesicle increased. Although phase variation did not affect sperm storage in the proximal part of seminal vesicle (*χ*^2^ = 0.00045826, *p* = 0.9829) (Table 4), it did affect sperm storage in the distal part of seminal vesicle (*χ*^2^ = 21.519381, *p* < 0.0001) (Table 4), suggesting that gregarious males are likely to have higher sperm levels in the seminal vesicle than solitary males.

### 3.3. Effects of Pheromones on Sperm Storage

Sperm distribution did not differ between adults exposed to pheromones from mature adults versus from immature nymphs at any adult age except for Day 3 (Table 5). Although neither type of pheromone affected sperm distribution in the vasa deferentia or seminal vesicles, age did affect sperm storage in male locusts exposed to both kinds of pheromone (Table 6). The number of folds in the seminal vesicle did not differ between the two pheromone treatments (Table 7).

**Figure 3:**
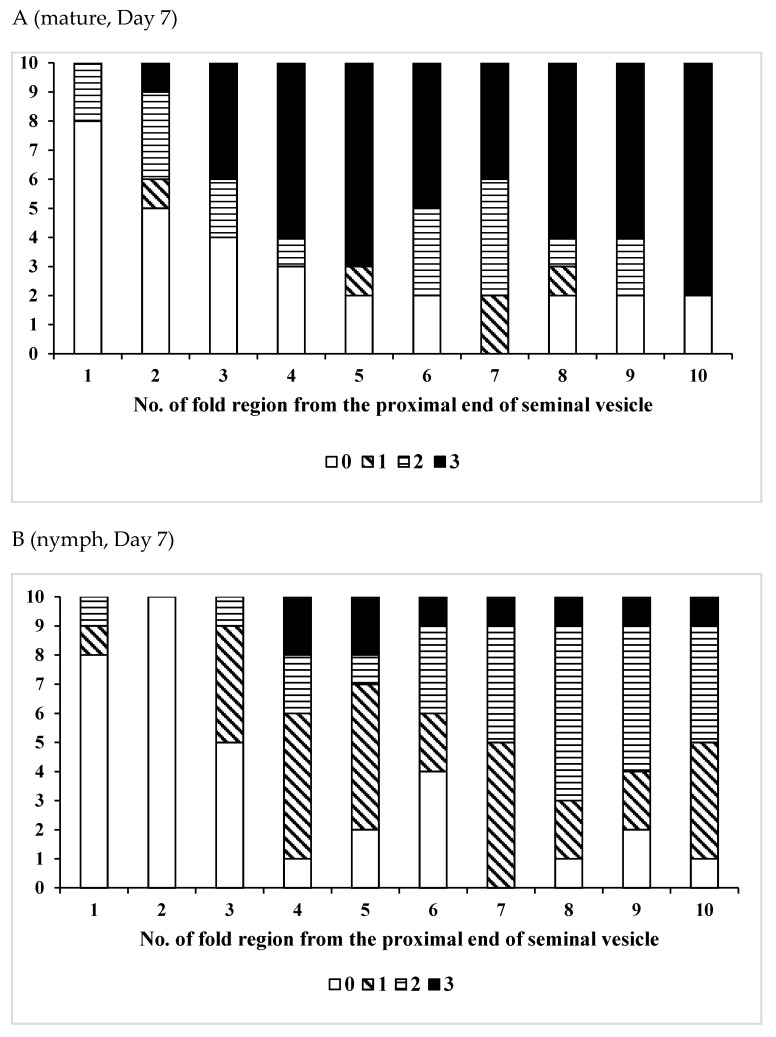

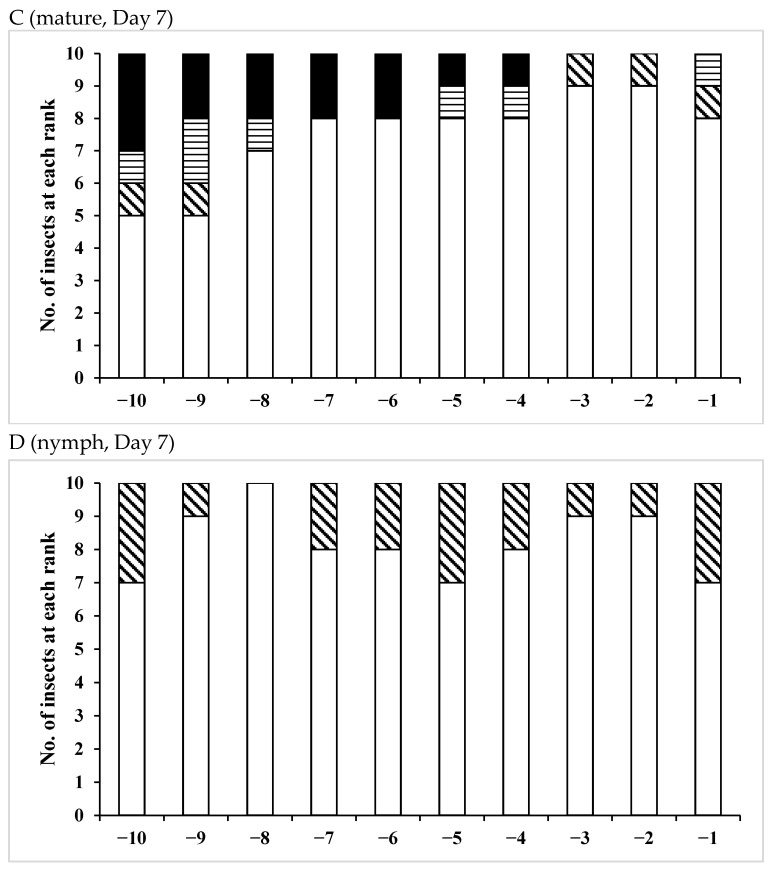

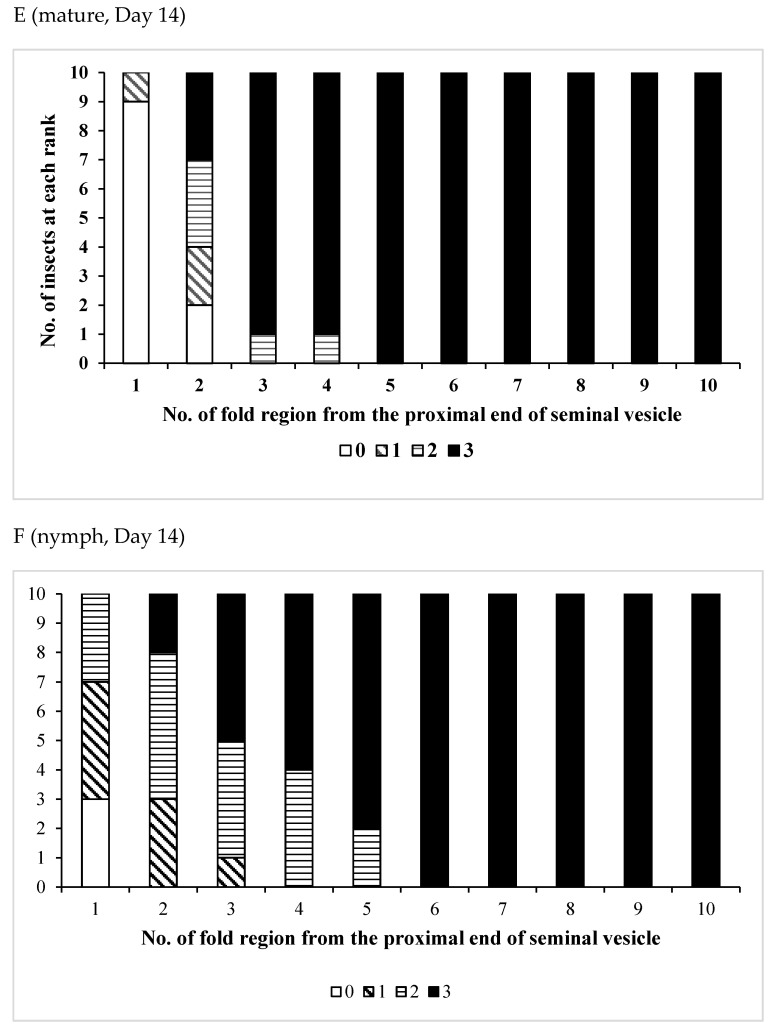

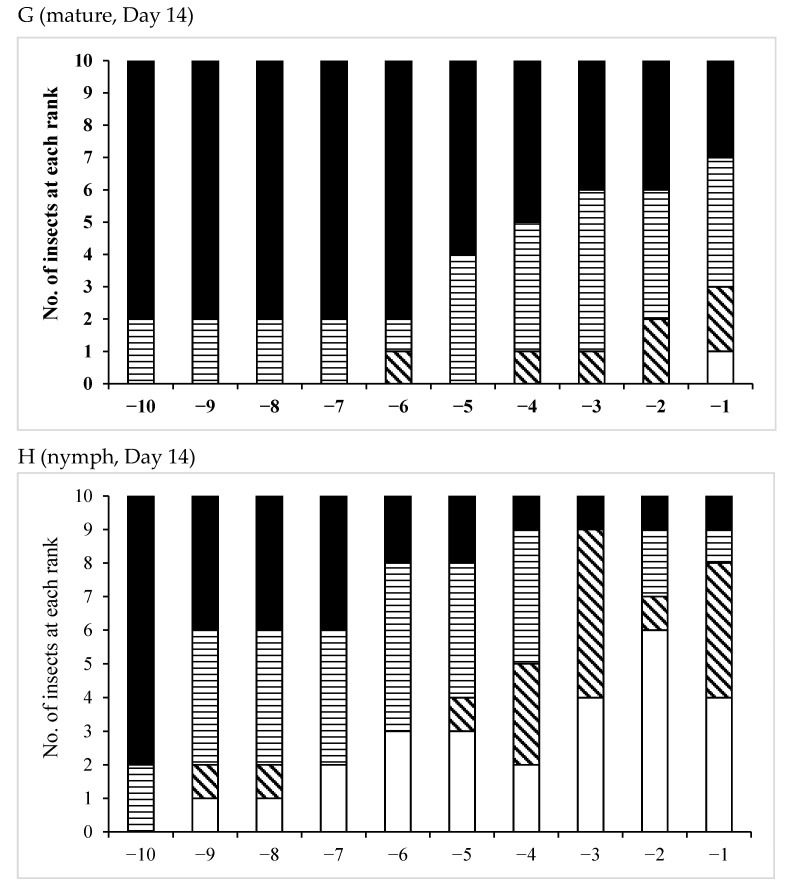

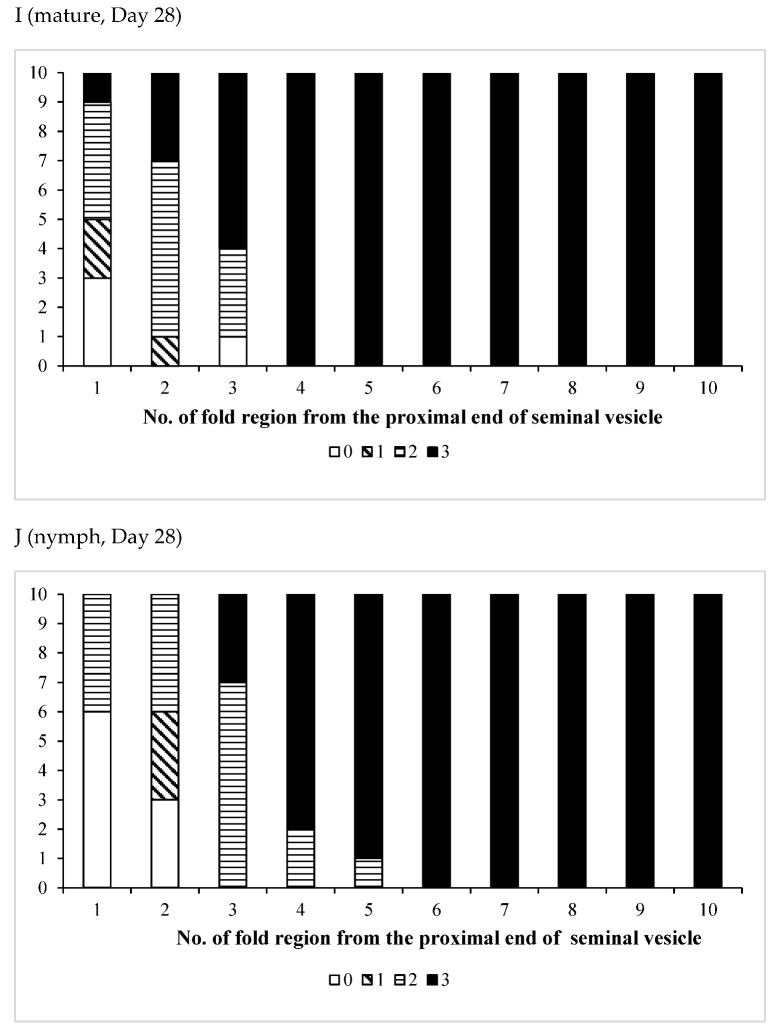

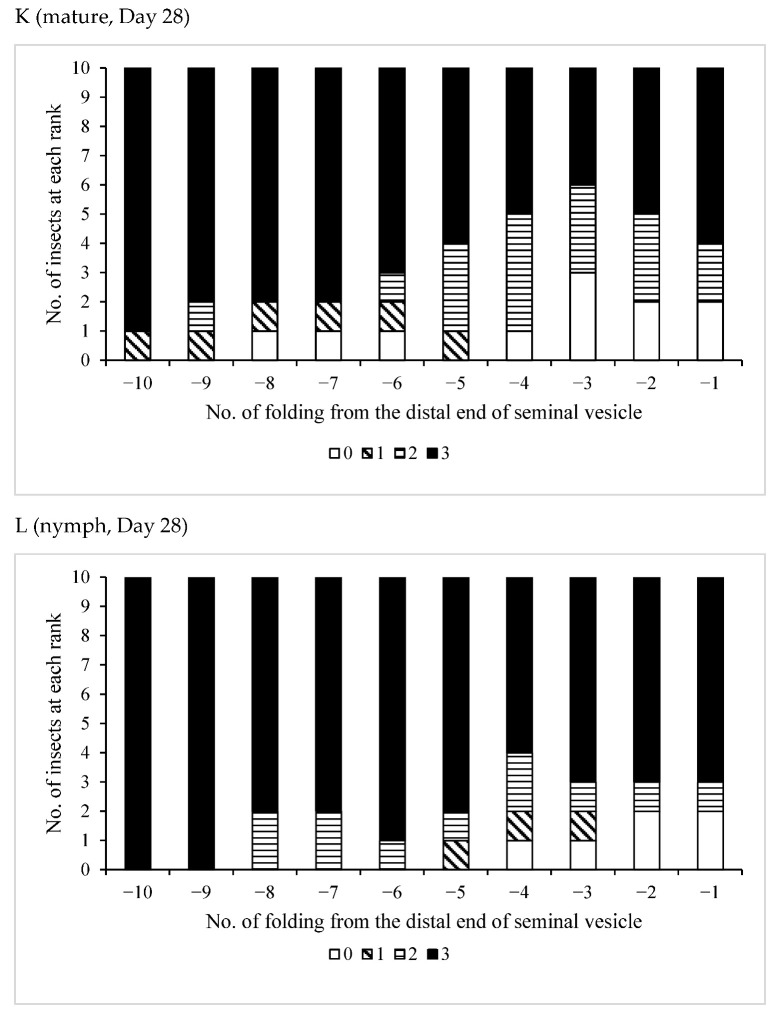
Distribution of sperm in the proximal part (**A**,**E**,**I**) or the distal part (**C**,**G**,**K**) of the seminal vesicle of the gregarious form, when immature adult locusts were influenced by the maturation accelerating pheromones, and that in the proximal part (**B**,**F**,**J**) or the distal part (**D**,**H**,**L**) of the seminal vesicle of the gregarious form of the desert locust, *Shistocerca gregaria*, when immature adult locusts were influenced by the maturation retarding pheromones. (**A**–**D**), (**E**–**H**), and (**I**–**L**) indicate Day 7, 14, and 28 of adult life, respectively. The number of sperm was classified into four categories by observing each fold region of the seminal vesicle under a microscope as follows; 0 = empty, 1 = several free sperm and/or <10 sperm bundles are seen in the fold (a few), 2 = ≥10 dispersed sperm bundles and/or intermittent sperm bundles mass (common), and 3 = many sperm bundles are packed tightly in the fold (many).

At 1 week after adult emergence, recipient males exposed to pheromones from mature adults had many sperm or sperm bundles in the proximal part (+4 to +10th fold regions) of the seminal vesicle, in 70 to 80% of individuals (Figure 3A). Additionally, at 1 week, most males (80–90%) lacked sperm in the distal part (−7 to −1st fold regions) of the seminal vesicle (Figure 3C). Additionally, at 2 weeks, except for the first two fold regions, many sperm bundles were found in the proximal part (+3 to +10th fold regions) of the seminal vesicle in all males (Figure 3E). Additionally, at 2 weeks, 90% of males had many sperm or sperm bundles in the distal part of the seminal vesicle, except for the last two fold regions (Figure 3G). At 4 weeks after adult emergence, all males had huge sperm bundles in the proximal part (+4 to +10th fold regions) of the seminal vesicle (Figure 3I). At the same time, there were many sperm bundles in the distal portion of the seminal vesicle in 40 to 90% of males (Figure 3K).

For males exposed to nymphal pheromones, at one week after adult emergence, 30% to 70% had many sperm in the proximal part of the seminal vesicle except for the first threefold regions (Figure 3B), but most (70 to 100%) had no sperm in the distal part of the seminal vesicle (Figure 3D). At two weeks after adult emergence, there were many sperm or sperm bundles in the proximal part (+3 to +10th fold regions) of the seminal vesicle in 90 to 100% of males (Figure 3F), but only in the −10th fold region of distal part were there many sperm bundles. The sperm quantity decreased with increasing distance toward the distal end (Figure 3H). Four weeks after adult emergence, many sperm bundles were found in the proximal part of the seminal vesicle (except for the first three fold regions) in 80 to 100% of males (Figure 3J), and also in the distal part (−10 to −1st fold region), there were many sperm bundles in 80 to 100% of males (Figure 3L).

As adult males aged, the quantity of sperm in their seminal vesicles increased. As sperm migration occurred from the proximal end to the center of the seminal vesicle and from the center to the distal end, the sperm quantity also increased (Table 8). Sperm quantity of males exposed to the pheromone of mature adults tended to be greater than that of males exposed nymphal pheromones. As adult males aged, the difference in sperm storage between males exposed to mature adults and nymphal pheromones disappeared, as male locusts which received maturation retarding pheromones also became sexually mature.

## 4. Discussion

The formation of mature sperm in the desert locust occurs shortly after adult emergence [37,52], although spermiogenesis starts before emergence in the Moroccan locust *Dociostaurus maroccanus* (Thunberg) [53]. After that, spermiogenesis lasts for at least one month under laboratory conditions, and probably continues throughout adult life. Sperm movement from the testis to the seminal vesicle via the vas deferens started 3 days after adult emergence in the earlier developing males [54], similar to this study, although the commencement of sperm migration differed among individuals and temperatures considerably. The supply of sperm from the testis to the seminal vesicle also seems to last for a long time due to the high ability of production and delivery of sperm from the testis, which is supported by the fact that most mature males examined had many sperm in the vas deferens at all ages. Irrespective of phase (solitary or gregarious), in young (sexually immature) individuals, sperm were observed only in the proximal (anterior) part of the seminal vesicle and were found in the distal (posterior) part of the seminal vesicle only in the older (sexually mature) locusts (Figure 2).

It is generally believed that male grasshoppers or locusts ejaculate only a small proportion of sperm in the seminal vesicle into females during mating, as the migratory grasshopper, *Melanoplus sanguinipes* (F.) can copulate several times on a single day, and on each occasion transfer several spermatophores [55,56]. On the day of male emergence, there were no sperm in the vas deferens and seminal vesicle of the desert locust. One week after adult emergence, all males had the sperm in the seminal vesicle, especially in the anterior portion of seminal vesicle. The older the adult male, the deeper the sperm had migrated in the seminal vesicle. These results strongly suggest that the sperm migrated from the proximal part to the distal part of seminal vesicle. This also suggests that adult males probably use the newly transferred sperm located near the proximal part of the seminal vesicle for mating, and thus the sperm located in the distal part of the seminal vesicle remains to be used in the subsequent mating. Indeed, many sperm bundles were observed in the seminal vesicle after mating (Hiroyoshi, unpublished observation). Therefore, this may affect fertility or sperm competition, as the sperm located in the distal part of seminal vesicle may be too old. Recent studies suggest that aged individuals have dead sperm in the spermatheca [57,58,59,60]. Although dead versus alive of sperm were not distinguished in our analysis of the desert locust, it seems likely that old sperm might reduce female fertility. This could be related to the structure of the seminal vesicle (very long and slender).

The present study investigated the effects of phase variation and pheromones on the pattern of sperm distribution in the seminal vesicle of the desert locust. Overall, sperm accumulation in the seminal vesicle in the solitary locusts tended to be delayed compared to gregarious locusts. Given that our solitary locusts were held singly, but our gregarious locusts were reared as groups, both physical contact and exposure to external pheromones or other factors may have been involved in this difference in the timing of sperm accumulation. This point should be examined in the future.

The effects of pheromones on the patterns of sperm accumulation in the seminal vesicle were evident within one week after adult emergence (Figure 3). By week 2 after adult emergence, the recipient males were exposed to the maturation accelerating pheromone from mature locusts, and there were many sperm bundles (Figure 3). In contrast, in males exposed to maturation retarding pheromone from the nymphs, there were relatively few sperm bundles in the seminal vesicle during the same period (Figure 3).

It has been thought that changes of body coloration and sexual maturation of males are regulated, at least partially, by the corpora allata juvenile hormones [8,28,39,43,61,62]. Several reports have examined the relationship between corpora allata activity and components or quantity of proteins in the male accessory glands of *M. sanguinipes*. Protein synthesis in the male accessory glands is promoted by transplantation of active corpora allata or administration of juvenile hormone, but these procedures do not affect the seminal vesicle [49,63,64]. Similarly, accumulation of protein in the accessory gland of *M*. *sanguinipes* is affected by the allatectomy, although the seminal vesicles affected only slightly [65]. One may argue that the process of sperm storage is not related to alternation of maturation rate of adult males due to the pheromones or phase variation or the activity of corpora allata. However, the present study indicated that the pheromones derived from mature adult males promoted sperm storage, and thus the above proposition is not supported by that evidence. Although the mechanisms underlying sperm migration in locusts are unclear, the activity of corpora allata, which is closely related to sexual maturation, or other factor(s) might be essential to consider when determining the mechanism of sperm storage. Ecdysteroids affect sperm migration from the testis to the vas deferens in Lepidoptera [66] and ecdysteroid levels in the male accessory gland in just-mated males are greater than those in unmated ones [67]. Consequently, further clarifying the action and influence of ecdysteroids upon sperm storage in locusts is needed.

## 5. Conclusions

The present research suggests that sexual maturation induced by the maturation accelerating pheromone appears to be related to promotion of sperm storage in the seminal vesicle of this locust. Although sperm storage increases with adult age regardless of pheromones or phase variation, the difference in sperm quantity disappeared. Adult age, temperatures, phase variation, and pheromones are essential for male sperm storage for this locust.

## Figures and Tables

**Table 1:** Comparison of sperm distribution in the vasa deferentia and seminal vesicle between solitary (S) and gregarious (G) adult males in the desert locust *Schistocerca gregaria*.

Age (Days)/Phase	No. of Insects Used	Vas Deferens −	Presence of Sperm +	Seminal Vesicle −	+
0					
/Solitary	8	8	0	8	0
/Gregarious	6	6	0	6	0
5 /Gregarious 7	5	4	1	4	1
/Solitary	11	1	10	1	10
/Gregarious	14	2	12	1	13
14					
/Solitary	10	0	10	0	10
/Gregarious	8	1	7	0	8
28					
/Solitary	10	2	8	0	10
/Gregarious	9	0	9	0	9

**Table 2:** The results of ordinary logistic regression analysis on the effects of phase variation and adult age on sperm distribution in the vas deferens and seminal vesicle of the desert locust *Schistocerca gregaria*.

Organ/Factor	Parameter	*df*	χ^2^	*p*-Value
Vas deferens/Age	1	1	14.8269711	0.0001 *
/Phase	1	1	0.97583528	0.3232
/Age × Phase	1	1	1.11782763	0.2904
Seminal vesicle/Age	1	1	27.8032765	<0.0001 *
/Phase	1	1	0.00023428	0.9878
/Age × Phase	1	1	0.06424243	0.7999

The asterisk indicates the significant difference at *p* < 0.05.

**Table 3:** The number of fold regions of the seminal vesicle compared between solitary (S) and gregarious (G) phases in the desert locust *Schistocerca gregaria*.

Age(Days)/Phase	No. of Insects Used	No of Fold Region Range	Average ± SD	*p* (S vs. G)
0				
/Solitary	10	26–41	33.7 ± 5.12	0.0110 *
/Gregarious	6	19–34	25.3 ± 5.09	
7				
/Solitary	11	27–37	32.5 ± 4.06	0.0217 *
/Gregarious	10	23–36	27.5 ± 4.35	
14				
/Solitary	10	28–38	32.3 ± 2.91	0.0068 *
/Gregarious	7	22–31	26.3 ± 3.55	
28				
/Solitary	15	22–38	31.7 ± 5.12	0.0548
/Gregarious	6	24–32	27.3 ± 2.94	

The asterisk indicates the significant difference at *p* < 0.05.

**Table 4:** The results of ordinary logistic regression analysis on the effects of phase variation and adult age on sperm storage in the desert locust *Schistocerca gregaria*, when sperm accumulation was counted from the proximal end or distal end of the folded seminal vesicle.

Factor	Parameter	*df*	χ^2^	*p* Value
Phase	1	1	0.00045826	0.9829
Age	1	1	356.79833	<0.0001 *
Phase × Age	1	1	0.74347209	0.3886
Fold region from proximal end (FPE)	1	1	200. 358912	<0.0001 *
Phase × FPE	1	1	10.3517704	0.0013 *
Age × FPE	1	1	38.0121077	<0.0001 *
Phase × Age × FPE	1	1	4.65029832	0.0310 *
Phase	1	1	21.519381	<0.0001 *
Age	1	1	424.940556	<0.0001 *
Phase × Age	1	1	0.111168	0.7388
Fold region from distal end (FDE)	1	1	18.5427795	<0.0001 *
Phase × FDE	1	1	0.87103289	0.3507
Age × FDE	1	1	0.76035844	0.3832
Phase × Age × FDE	1	1	0.16038184	0.6888

The asterisk indicates the significant difference at *p* < 0.05.

**Table 5:** Comparison of sperm distribution in the vas deferens and seminal vesicle between adult males that received the pheromone from mature adult males (Mature) and those from nymphs (Nymph) in the desert locust *Schistocerca gregaria*.

Age (Days)/Pheromone	No. of Insects Used	Vas Deferens −	Presence of Sperm +	Seminal Vesicle −	+
3					
/Mature	10	4	6	5	5
/Nymph	9	8	1	7	2
7					
/Mature	15	3	12	2	13
/Nymph	14	2	12	0	14
14					
/Mature	12	0	12	0	12
/Nymph	13	0	13	0	13
28					
/Mature	11	0	11	0	11
/Nymph	13	1	12	0	13

**Table 6:** The results of ordinary logistic regression analysis on the effects of pheromones and adult age on sperm distribution in the vas deferens and seminal vesicle of the desert locust *Schistocerca gregaria*.

Organ/Factor	Parameter	*df*	χ^2^	*p*-Value
Vas deferens/Age	1	1	16.5741729	0.0001 *
/Pheromone	1	1	0.59408121	0.4408
/Age × Pheromone	1	1	0.00217653	0.9628
Seminal vesicle/Age	1	1	22.5726214	0.0001 *
/Pheromone	1	1	0.03213105	0.8577
/Age × Pheromone	1	1	0.41065097	0.5216

The asterisk indicates the significant difference at *p* < 0.05.

**Table 7:** The number of fold region of seminal vesicle compared between adult males that received the pheromone from mature adults (Mature) and from nymphs (Nymph) in the desert locust *Schistocerca gregaria*.

Age (Days)/Phase	No. of Insects Used	No of Fold Region Range	Average ± SD	*p* (S vs. G)
3				
/Mature	9	17–35	25.6 ± 6.15	0.7962
/Nymph	9	20–33	25.8 ± 4.32	
7				
/Mature	15	18–35	25.3 ± 4.88	0.5537
/Nymph	14	20–32	26.1 ± 3.68	
14				
/Mature	12	21–29	26.7 ± 2.10	0.1197
/Nymph	13	24–28	25.8 ± 1.57	
28				
/Mature	10	24–35	30.5 ± 3.44	0.1604
/Nymph	13	25–33	28.7 ± 2.50	

**Table 8:** The results of ordinary logistic regression analysis on the effects of pheromone emitted from nymph or adult and adult age on sperm storage in the desert locust *Schistocerca gregaria*, when sperm accumulation was counted from the proximal end or distal end of the folded seminal vesicle.

Factor	Parameter	*df*	χ^2^	*p* Value
Pheromone	1	1	9.02784363	0.0027 *
Age	1	1	244.925256	<0.0001 *
Pheromone × Age	1	1	1.10510193	0.2931
Fold region from proximal end (FPE)	1	1	350.413422	<0.0001 *
Pheromone × FPE	1	1	3.07012175	0.0797 *
Age × FPE	1	1	126.245783	<0.0001 *
Pheromone × Age × FPE	1	1	0.2856723	0.5930
Pheromone	1	1	4.19837727	0.0405 *
Age	1	1	288.467967	<0.0001 *
Pheromone × Age	1	1	13.5989836	0.0002 *
Fold region from distal end (FDE)	1	1	53.2676067	<0.0001 *
Pheromone × FDE	1	1	0.27928405	0.5972
Age × FDE	1	1	7.75091207	0.0054 *
Pheromone × Age × FDE	1	1	2.15718014	0.1419

The asterisk indicates the significant difference at *p* < 0.05.

## Data Availability

Not applicable.
